# Effect of the Compaction and the Size of DNA on the Nuclear Transfer Efficiency after Microinjection in Synchronized Cells

**DOI:** 10.3390/pharmaceutics7020064

**Published:** 2015-06-09

**Authors:** Hidetaka Akita, Dai Kurihara, Marco Schmeer, Martin Schleef, Hideyoshi Harashima

**Affiliations:** 1 Faculty of Pharmaceutical Science, Hokkaido University, Kita-12, Nishi-6, Kita-ku, 060-0812 Sapporo, Japan; E-Mail: dai.kurihara@nipponkayaku.co.jp; 2 PlasmidFactory GmbH & Co. KG. Meisenstraße 96, D-33607 Bielefeld, Germany; E-Mails: marco.schmeer@plasmidfactory.com (Marc.S.); martin.schleef@plasmidfactory.com (Mart.S.)

**Keywords:** DNA, nuclear transfer, size, microinjection

## Abstract

The nuclear transfer process is one of the critical rate-limiting processes in transgene expression. In the present study, we report on the effect of compaction and the size of the DNA molecule on nuclear transfer efficiency by microinjection. A DNA/protamine complex- or variously-sized naked DNA molecules were injected into the cytoplasm or nucleus of synchronized HeLa cells. To evaluate the nuclear transfer process, a nuclear transfer score (NT score), calculated based on transgene expression after cytoplasmic microinjection divided by that after nuclear microinjection, was employed. The compaction of DNA with protamine decreased the NT score in comparison with the injection of naked DNA when the N/P ratio was increased to >2.0. Moreover, when naked DNA was microinjected, gene expression increased in parallel with the size of the DNA in the following order: minicircle DNA (MC07.CMV-EGFP; 2257 bp) > middle-sized plasmid DNA (pBS-EGFP; 3992 bp) > conventional plasmid DNA (pcDNA3.1-EGFP; 6172 bp), while the level of gene expression was quite comparable among them when the DNAs were injected into the nucleus. The above findings suggest that the intrinsic size of the DNA molecule is a major determinant for nuclear entry and that minicircle DNA has a great advantage in nuclear transfer.

## 1. Introduction

The nuclear delivery of plasmid DNA (pDNA) is a crucial rate-limiting process for successful gene expression. The most typical example in support of this theory is that transgene expression is drastically enhanced after cells undergo mitosis, when the nuclear membrane is diminished [1,2,3,4]. Furthermore, a comparison of the dose-response curves for transgene expression after the cytoplasmic and nuclear microinjection of naked pDNA indicates that >1% of the pDNA actually reaches the nucleus [5].

In non-dividing cells, the mutual transport of endogenous substances between the cytoplasm and nucleus occurs largely via the nuclear pore complex (NPC). One of the most important characteristics of NPC-dependent transport is that the threshold size of the molecules that can freely diffuse through the NPC is <9 nm [6]. The mechanism for selective nuclear transport has been also investigated in the past decades. The nuclear localization signal (NLS)-dependent nuclear import of proteins is the best characterized example of this [7]. Endogenous or chemically-modified NLSs are recognized by karyopherins (*i.e.*, importin α and/or β), and the cargo is then allowed to pass though the nuclear pore, with the aid of interactions with hydrophobic phenylalanine-glycine (FG)-rich domains inside the NPC [8,9,10]. Another type of nuclear import carrier (*i.e.*, Hikeshi) that is not a member of the conventional importin family has recently been identified as the nuclear import machinery of Hsp70s [11]. Furthermore, the karyopherin-independent transport of amphiphilic proteins (*i.e.*, β-catenin) has also been reported [12].

In light of these studies, pDNA was compacted with various types of materials that were modified with an NLS (*i.e.*, poly-l-lysine modified with an NLS [13,14]) via electrostatic interactions. Alternatively, peptides that are fused with an NLS or that intrinsically function as an NLS (*i.e.*, protamine [15,16], a tetramer of NLSs [17] and NLS-μ [18]) were also used as a compaction agent for the pDNA. In parallel, Dean and collaborators revealed that certain specific sequences in pDNA [19,20] such as the NF-κB binding sequence [21,22] or the SV40 enhancer [19,20,23] allow the binding of nuclear transcription factors in the cytosol, and then, the nuclear import is assisted by cytoplasmic cofactors (*i.e.*, importin β1, importin 7 and the small guanosine triphosphatase Ran) [24] and microtubule-dependent transport [25]. Since most previous studies used dividing cells, the issue of which strategy is the better choice for achieving the nuclear delivery of pDNA in non-dividing cells remains elusive.

Other questions that need to be clarified are the effect of size on the nuclear transport process in non-dividing cells. Wolff and co-workers investigated the effect of pDNA size on nuclear transport activity using digitonin-permeabilized cells [26,27]. Short DNA (<200 bp) fragments were readily imported into the nucleus. While an NLS-dependent enhancement in nuclear transport of up to 3 kb was achieved, it still remains to be clarified whether the size of pDNA is a key factor for nuclear entry in the larger size range, since the NLS-dependent transport of molecules is, in turn, limited by its threshold size (<39 nm) [28].

The object of this study was to examine the effect of compaction, and the size of DNA on nuclear transfer efficiency by microinjection into synchronized cells (non-dividing cells) using GFP as a marker gene. Transgene expression after cytoplasmic microinjection (E(cyt)) is highly dependent on intra-nuclear transcription efficiency, as well as nuclear transfer efficiency. In contrast, the corresponding value after nuclear microinjection (E(nuc)) represents the efficiency of nuclear transcription. Taking these into consideration, we introduced a nuclear transfer score (NT score), denoted as E(cyt) divided by E(nuc), as an index for comparing the nuclear transfer efficiency [15,18]. The first objective of the present study was to analyze the effect of compaction with protamine on the nuclear transport efficiency of DNA. As described below, our analyses revealed that compaction resulted in a decrease in nuclear transport. Thus, the second goal was to clarify the effect of the size of the DNA molecule on nuclear transfer efficiency when the DNA was injected in naked form. To ultimately shorten the size, minicircle DNA, in which the backbone region was completely removed, was used in this analysis [29,30].

## 2. Materials and Methods

### 2.1. General

HeLa cells were obtained from the RIKEN Cell Bank (Tsukuba, Japan). pcDNA3.1-EGFP (6172 bp) was prepared, as reported previously [15]. To prepare the pDNA, pBS-EGFP (3992 bp), the StuI site was preliminarily inserted in the multiple cloning site (MCS) in pBlueScript SKII(+). The EGFP-expression cassette was obtained by the digestion of pEGFP-N1 (Clontech, Palo Alto, CA, USA) with AflIII/AflII and then blunt-ended by KOD DNA polymerase (TOYOBO, Osaka, Japan). The fragment was inserted into the StuI site of pBlueScript SKII(+). Minicircle DNA (MC07.CMV-EGFP) was supplied from PlasmidFactory GmbH & Co. KG (Bielefeld, Germany). Protamine sulfate was obtained from CALBIOCHEM (Ishikari, Japan) in purified form. Before use, the protamine solution was filtered through the cellulose acetate filter (DISMIC-13cp: 0.2 µm pore size) that was obtained from ADVANTECH (Chiba, Japan). Tetramethylrhodamine-labeled dextran (RhoDex: MW 70,000) was purchased from Life Technologies Japan. Ltd. (Tokyo, Japan).

### 2.2. Preparation of Protamine/pDNA Complex

To form a DNA complex with protamine, 20 µL of a minicircle DNA solution (MC07.CMV-EGFP; 0.1 µg/µL in H_2_O) were added dropwise to 50 µL of protamine solution under vortexing 5 times (100 µL in total) at approximately 20 s-intervals. The concentration of the protamine (*C*_protamine_) at various amine/phosphate ratios (N/P) was calculated using the following equation:

N/P ratio = (*C*_protamine_ × *n*_cation_/MW_protamine_) / (*C*_DNA_ × *n*_bp_ × 2/MW_DNA_) (1)

where *n*_cation_ and *n*_bp_ denote the number of lysine and arginine residues in protamine and the number of base pairs in the minicircle DNA, respectively. MW_protamine_ and MW_DNA_ denote the molecular weight of the polycation (protamine: 4250), and the whole DNA that can be calculated based on the assumption that an average molecular weight of one nucleotide is 308. *C*_DNA_ denotes the concentration of minicircle DNA (0.1 µg/µL). The diameter of the particles was determined using an electrophoretic light-scattering spectrophotometer (Zetasizer; Malvern Instruments Ltd., Malvern, UK).

### 2.3. Cells, Cell Culture and Cell Cycle Synchronization

HeLa (human cervical carcinoma) cells were maintained in Dulbecco’s Modified Eagle’s Medium (DMEM) supplemented with 10% fetal bovine serum, penicillin (100 units/mL) and streptomycin (100 μg/mL). These cells were cultured under an atmosphere of 5% CO_2_/air at 37 °C. Cell-cycle synchronization was performed by culturing the cells in a medium containing hydroxyurea (2.5 mM).

### 2.4. Microinjection

Microinjection was carried out via the use of a semi-automatic injection system (Eppendorf Transjector 5246) attached to an Eppendorf Micromanipulator 5171 (Eppendorf, Hamburg, Germany) mounted on an inverted microscope (Axiovert 100; Carl Zeiss Co. Ltd., Jena, Germany). Cells were seeded on a glass-base dish (Iwaki, Osaka, Japan) 2 days before microinjection. In the case of cell-cycle synchronization, the cells were incubated with hydroxyurea for 18 h before microinjection, and all subsequent steps were performed in the presence of 2.5 mM hydroxyurea. DNAs were diluted to 3.32 pmol/mL and 33.2 fmol/mL with a 0.5% RhoDex/H_2_O solution as a microinjection marker, and then microinjected into the cytoplasm and nuclei of the cells, respectively. Nuclear or cytoplasmic injections were performed under an injection pressure of 50 hPa and a maintenance pressure of 30 hPa, with an injection time of 0.2 s. We assumed that approximately 0.5 pL of the samples were injected per 1 injection. The DNA samples were diluted so that 1000 and 10 copies of DNA were injected into the cytoplasm and nucleus, respectively. Just after microinjection, RhoDex-positive cells were counted using a fluorescence microscope (Axioplan 2; Zeiss). At 24 h post-injection, the number of cells expressing EGFP was counted, and the ratio of EGFP-positive cells to RhoDex-positive cells was calculated.

## 3. Results and Discussion

### 3.1. Synchronization of the Cells

**Figure 1:**
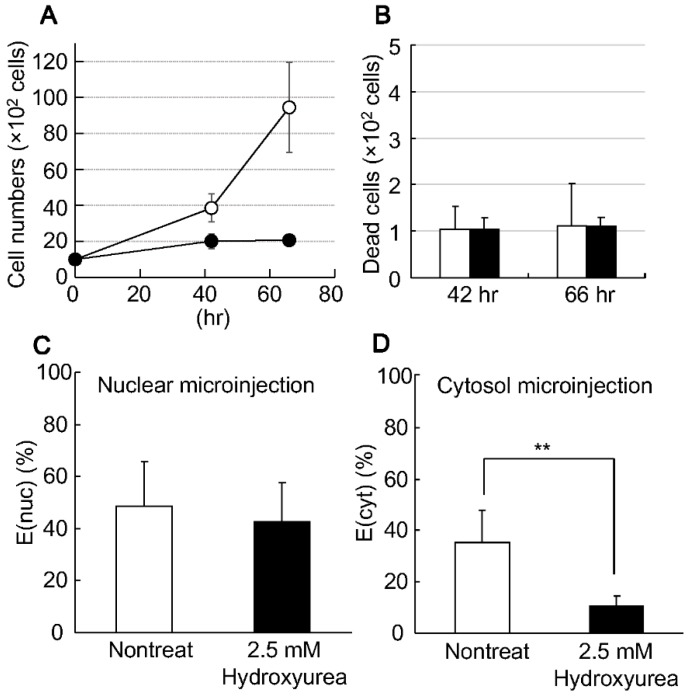
Synchronization of HeLa cells by hydroxyurea. Cell growth (**A**) and cell death (**B**) was monitored by counting the number of HeLa cells. The cells were cultured in the presence (closed) or absence (open) of 2.5 mM of hydroxyurea. The number of dead cells was counted by staining the cells with trypan blue. Gene expression of the EGFP after the microinjection of MC07.CMV-EGFP in the nucleus (**C**) or cytoplasm (**D**) of the non-treated cells (open) or synchronized cells (closed). Data are represented as the mean ± S.D. of triplicate experiments. The statistical differences were determined by the one-way ANOVA followed by Student’s *t*-test (**, *p* < 0.01). E(nuc), transgene expression after nuclear microinjection; E(cyt), transgene expression after cytoplasmic microinjection.

In the present study, synchronized HeLa cells were prepared by treatment with 2.5 mM of hydroxyurea as a model of non-dividing cells. The inhibition of mitosis was confirmed by monitoring the time-dependent cell growth. While the cells grew rapidly in normal medium, growth was completely blocked in the presence of hydroxyurea (Figure 1A). In this case, the number of the dead cells that were stained by trypan blue was comparable regardless of the presence or absence of hydroxyurea (Figure 1B). Thus, the inhibition of cell growth is due to the prevention of mitosis, not cell damage.

To investigate whether the nuclear import of DNA was inhibited when the growth became slow, the transgene expression of DNA (expression of EGFP) was evaluated after the microinjection. The E(nuc) values was comparable even when the nucleus was injected with 10 copies of MC07.CMV-EGFP (Figure 1C) in synchronized or non-synchronized cells. Thus, transcription efficiency was not affected by the hydroxyurea treatment. In contrast, the E(cyt) was impaired by the synchronization (Figure 1D). These results indicate that the hydroxyurea treatment blocked the NPC-independent nuclear delivery process; non-specific nuclear delivery concomitant with the temporal collapse of the nuclear membrane structure at mitosis. In other words, synchronized cells can be considered to be useful for evaluating NPC-dependent nuclear delivery.

### 3.2. Effect of DNA Compaction with Protamine on Nuclear Delivery Efficiency

**Figure 2:**
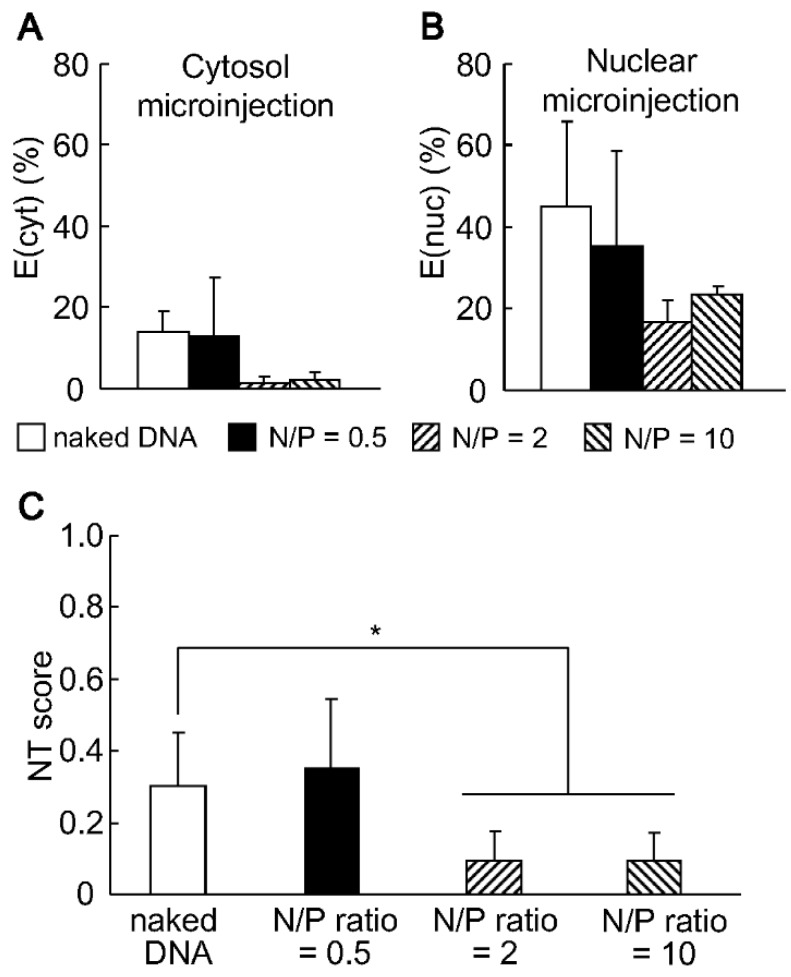
EGFP expression after the cytoplasmic and nuclear microinjection of protamine/DNA complexes. The minicircle DNA (MC07.CMV-EGFP) was compacted with protamine at N/P ratio of 0.5, 2 and 9. One thousand copies and 10 copies of DNA were injected into the cytoplasm (**A**) and the nucleus (**B**) with 0.5% RhoDex, respectively. At 24 h post injection, the ratio of GFP-positive cells to RhoDex-positive cells was calculated. (**C**) The nuclear transfer (NT) scores were calculated as the percent of EGFP-expression after cytoplasmic microinjection (E(cyt)) divided by that after nuclear microinjection (E(nuc)). Vertical bars indicate the standard deviation of triplicate experiments. The statistical differences were determined by one-way ANOVA followed by the Student–Newman–Keuls test. (*, *p* < 0.05).

We previously reported that the compaction of pDNA with protamine enhanced the efficiency of gene expression in a N/P ratio-dependent manner after microinjecting dividing HeLa cells [15]. To investigate whether this enhanced effect can be reproduced in non-dividing cells, the minicircle DNA was complexed with protamine at N/P ratios of 0.5, 2 and 10 and then injected into the synchronized cells. The size of the particles gradually decreased from approximately 100 to 70 nm when the N/P ratio was increased (Table 1), owing to the extensive condensation of DNA with protamine. Unexpectedly, the compaction of minicircle DNA resulted in a drastic decrease in the E(cyt) value when the DNAs were compacted at a N/P ratio of >2.0, while the values were comparable at a N/P ratio of 0.5 (Figure 2A).

**Table 1:** Sizes of protamine/DNA complexes.

N/P Ratio	Diameter (nm)
0.5	102 ± 2
2	83.6 ± 12.5
10	72.9 ± 7.9

Meanwhile, the E(nuc) values gradually, but slightly, decreased (Figure 2B). The access of transcriptional factors to the DNA might have been prevented by the condensation. The calculated NT score indicated that the condensation of DNA resulted in a decreased nuclear transfer efficiency (Figure 2C).

The most controversial result is that the effect of compaction with protamine was completely opposite in dividing cells compared to synchronized cells; a previous study indicated that compaction enhanced the nuclear transfer in dividing cells [15], whereas it was decreased in synchronized cells. While conventional confocal laser scanning microscopy observations in the *Z*-axis are not sufficient to conclude that the DNA was actually transported to the nucleus, previous microscopic observations of dividing cells revealed that the pDNA/protamine complex was actually detected in the nuclear peripheral region [15]. Thus, the nuclear entry of DNA was accelerated at the mitosis phase once it was concentrated at the nuclear periphery. In contrast, in the case of synchronized cells, the NPC is a specific nuclear entry pathway, since the nuclear membrane structure remains intact. Since the NPC can accept a rigid body with a size within 39 nm, tightly-condensed particles with a size of >70 nm would not be able to pass though the NPC, but would be able to gain access to the nuclear periphery. Collectively, the condensation of DNA has a negative effect on the nuclear transfer efficiency in non-dividing cells.

### 3.3. Effect of the Size of DNA on Nuclear Transfer Efficiency

**Figure 3:**
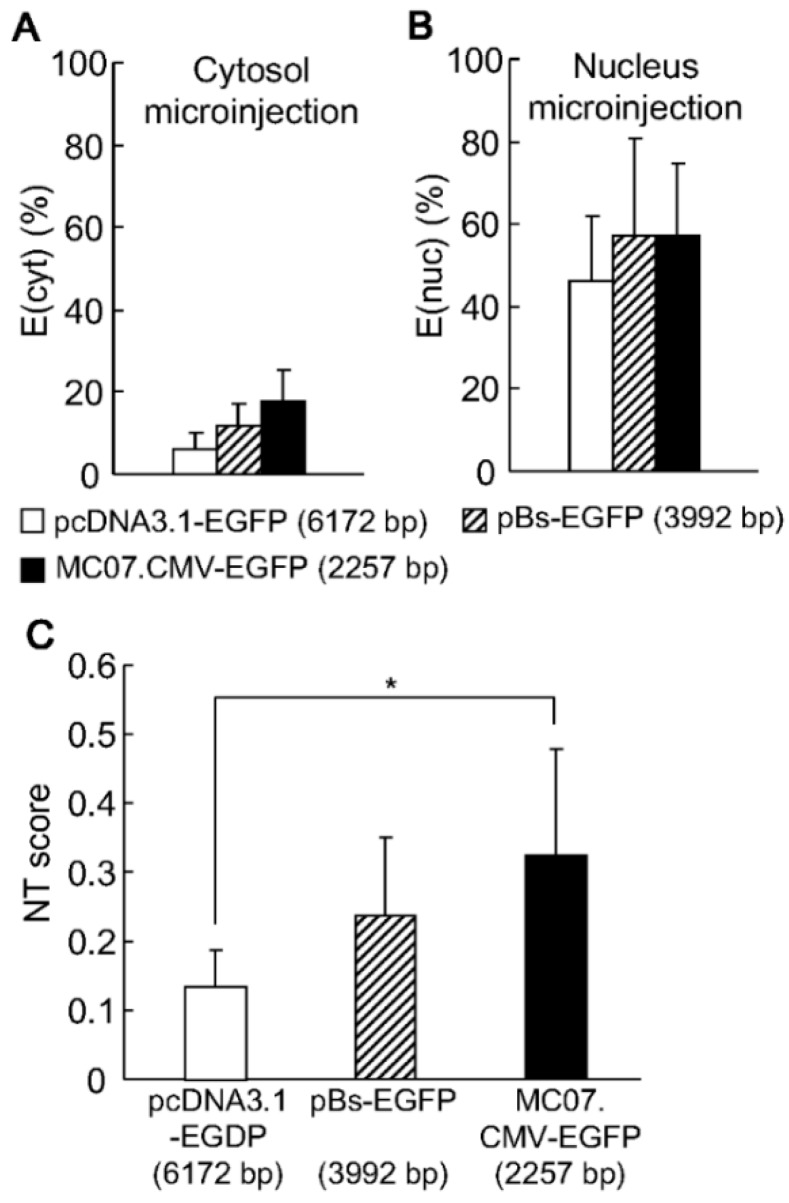
EGFP expression after the cytoplasmic and nuclear microinjection of naked DNA. One thousand copies and 10 copies of three types of DNAs (MC07.CMV-EGFP, pBS-EGFP and pcDNA3.1-EGFP) were injected into the cytoplasm (**A**) and nucleus (**B**) with 0.5% RhoDex. At 24 h post injection, the ratio of GFP-positive cells to RhoDex-positive cells was calculated. (**C**) NT scores were calculated as the percent of EGFP-expression after cytoplasmic microinjection (E(cyt)) divided by that after nuclear microinjection (E(nuc)). Vertical bars indicate the standard deviation of triplicate experiments. The statistical differences were determined by one-way ANOVA followed by the Student–Newman–Keuls test (*, *p* < 0.05).

Finally, the effect of DNA size on nuclear transfer was evaluated. As described above, the compaction or condensation of minicircle DNA by protamine resulted in a decrease in nuclear delivery. Thus, in the following study, we compared the NT scores for three types of DNAs with various sizes (MC07.CMV-EGFP, 2257 bp; pBS-EGFP, 3992 bp; and pcDNA3.1-EGFP, 6172 bp) after their injection in naked form. Regarding the E(cyt), a shorter pDNA exhibited a higher transgene expression (Figure 3A). In contrast, the E(nuc) was comparable regardless of the type of DNA used (Figure 3B). As a consequence, the NTscore increased as the size of the DNA molecule became smaller (Figure 3C). Of note, the NTscore for the minicircle DNA (MC07.CMV-EGFP) was significantly higher than that for the conventional pDNA (pcDNA3.1-EGFP). The minicircle DNA had a shorter length since the backbone had been completely removed. Thus, the use of minicircle DNA has a great advantage in maximizing nuclear transfer efficiency.

## 4. Conclusions

Various types of polymers or liposomes can be utilized for the delivery of DNA. Based on the findings reported herein, we conclude that compaction impairs the nuclear delivery of DNA in synchronized cells. Thus, the compaction of DNA with a biodegradable material might be an elegant idea from the point of view of enhancing the nuclear delivery of DNA. In this case, a smaller-sized DNA molecule, ultimately a minicircle DNA is the best choice in terms of maximizing nuclear transfer efficiency.

## References

[1] Brunner S., Sauer T., Carotta S., Cotten M., Saltik M., Wagner E. (2000). Cell cycle dependence of gene transfer by lipoplex, polyplex and recombinant adenovirus. Gene Ther..

[2] James M.B., Giorgio T.D. (2000). Nuclear-associated plasmid, but not cell-associated plasmid, is correlated with transgene expression in cultured mammalian cells. Mol. Ther..

[3] Mortimer I., Tam P., MacLachlan I., Graham R.W., Saravolac E.G., Joshi P.B. (1999). Cationic lipid-mediated transfection of cells in culture requires mitotic activity. Gene Ther..

[4] Tseng W.C., Haselton F.R., Giorgio T.D. (1999). Mitosis enhances transgene expression of plasmid delivered by cationic liposomes. Biochim. Biophys. Acta.

[5] Pollard H., Remy J.S., Loussouarn G., Demolombe S., Behr J.P., Escande D. (1998). Polyethylenimine but not cationic lipids promotes transgene delivery to the nucleus in mammalian cells. J. Biol. Chem..

[6] Allen T.D., Cronshaw J.M., Bagley S., Kiseleva E., Goldberg M.W. (2000). The nuclear pore complex: Mediator of translocation between nucleus and cytoplasm. J. Cell Sci..

[7] Stewart M. (2007). Molecular mechanism of the nuclear protein import cycle. Nat. Rev. Mol. Cell Biol..

[8] Ben-Efraim I., Gerace L. (2001). Gradient of increasing affinity of importin beta for nucleoporins along the pathway of nuclear import. J. Cell Biol..

[9] Yoshimura S.H., Kumeta M., Takeyasu K. (2014). Structural mechanism of nuclear transport mediated by importin beta and flexible amphiphilic proteins. Structure.

[10] Yoshimura S.H., Otsuka S., Kumeta M., Taga M., Takeyasu K. (2013). Intermolecular disulfide bonds between nucleoporins regulate karyopherin-dependent nuclear transport. J. Cell Sci..

[11] Kose S., Furuta M., Imamoto N. (2012). Hikeshi, a nuclear import carrier for Hsp70s, protects cells from heat shock-induced nuclear damage. Cell.

[12] Kumeta M., Yamaguchi H., Yoshimura S.H., Takeyasu K. (2012). Karyopherin-independent spontaneous transport of amphiphilic proteins through the nuclear pore. J. Cell Sci..

[13] Chan C.K., Jans D.A. (1999). Enhancement of polylysine-mediated transferrinfection by nuclear localization sequences: Polylysine does not function as a nuclear localization sequence. Hum. Gene Ther..

[14] Chan C.K., Senden T., Jans D.A. (2000). Supramolecular structure and nuclear targeting efficiency determine the enhancement of transfection by modified polylysines. Gene Ther..

[15] Masuda T., Akita H., Harashima H. (2005). Evaluation of nuclear transfer and transcription of plasmid DNA condensed with protamine by microinjection: The use of a nuclear transfer score. FEBS Lett..

[16] Sorgi F.L., Bhattacharya S., Huang L. (1997). Protamine sulfate enhances lipid-mediated gene transfer. Gene Ther..

[17] Ritter W., Plank C., Lausier J., Rudolph C., Zink D., Reinhardt D., Rosenecker J. (2003). A novel transfecting peptide comprising a tetrameric nuclear localization sequence. J. Mol. Med..

[18] Akita H., Tanimoto M., Masuda T., Kogure K., Hama S., Ninomiya K., Futaki S., Harashima H. (2006). Evaluation of the nuclear delivery and intra-nuclear transcription of plasmid DNA condensed with μ (mu) and NLS-μ by cytoplasmic and nuclear microinjection: A comparative study with poly-L-lysine. J. Gene Med..

[19] Dean D.A. (1997). Import of plasmid DNA into the nucleus is sequence specific. Exp. Cell Res..

[20] Dean D.A., Dean B.S., Muller S., Smith L.C. (1999). Sequence requirements for plasmid nuclear import. Exp. Cell Res..

[21] Mesika A., Grigoreva I., Zohar M., Reich Z. (2001). A regulated, NFkappaB-assisted import of plasmid DNA into mammalian cell nuclei. Mol. Ther..

[22] Mesika A., Kiss V., Brumfeld V., Ghosh G., Reich Z. (2005). Enhanced intracellular mobility and nuclear accumulation of DNA plasmids associated with a karyophilic protein. Hum. Gene Ther..

[23] Wilson G.L., Dean B.S., Wang G., Dean D.A. (1999). Nuclear import of plasmid DNA in digitonin-permeabilized cells requires both cytoplasmic factors and specific DNA sequences. J. Biol. Chem..

[24] Miller A.M., Munkonge F.M., Alton E.W., Dean D.A. (2009). Identification of protein cofactors necessary for sequence-specific plasmid DNA nuclear import. Mol. Ther..

[25] Badding M.A., Vaughan E.E., Dean D.A. (2012). Transcription factor plasmid binding modulates microtubule interactions and intracellular trafficking during gene transfer. Gene Ther..

[26] Hagstrom J.E., Ludtke J.J., Bassik M.C., Sebestyen M.G., Adam S.A., Wolff J.A. (1997). Nuclear import of DNA in digitonin-permeabilized cells. J. Cell Sci..

[27] Ludtke J.J., Zhang G., Sebestyen M.G., Wolff J.A. (1999). A nuclear localization signal can enhance both the nuclear transport and expression of 1 kb DNA. J. Cell Sci..

[28] Pante N., Kann M. (2002). Nuclear pore complex is able to transport macromolecules with diameters of about 39 nm. Mol. Biol. Cell.

[29] Gaspar V., de Melo-Diogo D., Costa E., Moreira A., Queiroz J., Pichon C., Correia I., Sousa F. (2015). Minicircle DNA vectors for gene therapy: Advances and applications. Exp. Opin. Biol. Ther..

[30] Kay M.A., He C.Y., Chen Z.Y. (2010). A robust system for production of minicircle DNA vectors. Nat. Biotechnol..

